# Design, Development and Evaluation of rK28-Based Point-of-Care Tests for Improving Rapid Diagnosis of Visceral Leishmaniasis

**DOI:** 10.1371/journal.pntd.0000822

**Published:** 2010-09-14

**Authors:** Sowmya Pattabhi, Jacqueline Whittle, Raodoh Mohamath, Sayda El-Safi, Garner G. Moulton, Jeffrey A. Guderian, Danny Colombara, Asem O. Abdoon, Maowia M. Mukhtar, Dinesh Mondal, Javan Esfandiari, Shailendra Kumar, Peter Chun, Steven G. Reed, Ajay Bhatia

**Affiliations:** 1 Infectious Disease Research Institute, Seattle, Washington, United States of America; 2 Department of Microbiology and Parasitology, Faculty of Medicine, Khartoum University, Khartoum, Sudan; 3 Department of Epidemiology, School of Public Health, University of Washington, Seattle, Washington, United States of America; 4 Institute of Endemic Diseases, University of Khartoum, Khartoum, Sudan; 5 Laboratory Sciences Division, International Centre for Diarrhoeal Diseases Research, Dhaka, Bangladesh; 6 Chembio Diagnostic Systems, Inc., Medford, New York, United States of America; 7 Ease-Medtrend, Shanghai, People's Republic of China; Institut Pasteur de Tunis, Tunisia

## Abstract

**Background:**

Visceral leishmaniasis (VL) is diagnosed by microscopic confirmation of the parasite in bone marrow, spleen or lymph node aspirates. These procedures are unsuitable for rapid diagnosis of VL in field settings. The development of rK39-based rapid diagnostic tests (RDT) revolutionized diagnosis of VL by offering high sensitivity and specificity in detecting disease in the Indian subcontinent; however, these tests have been less reliable in the African subcontinent (sensitivity range of 75–85%, specificity of 70–92%). We have addressed limitations of the rK39 with a new synthetic polyprotein, rK28, followed by development and evaluation of two new rK28-based RDT prototype platforms.

**Methodology/Principal Findings:**

Evaluation of 62 VL-confirmed sera from Sudan provided sensitivities of 96.8% and 93.6% (95% CI = K28: 88.83–99.61%; K39: 84.30–98.21%) and specificities of 96.2% and 92.4% (95% CI = K28: 90.53–98.95%; K39: 85.54–96.65%) for rK28 and rK39, respectively. Of greater interest was the observation that individual VL sera with low rK39 reactivity often had much higher rK28 reactivity. This characteristic of the fusion protein was exploited in the development of rK28 rapid tests, which may prove to be crucial in detecting VL among patients with low rK39 antibody levels. Evaluation of two prototype lateral flow-based rK28 rapid tests on 53 VL patients in Sudan and 73 VL patients in Bangladesh provided promisingly high sensitivities (95.9% [95% CI = 88.46–99.1 in Sudan and 98.1% [95% CI = 89.93–99.95%] in Bangladesh) compared to the rK39 RDT (sensitivities of 86.3% [95% CI = 76.25–93.23%] in Sudan and 88.7% [95% CI = 76.97–95.73%] in Bangladesh).

**Conclusions/Significance:**

Our study compares the diagnostic accuracy of rK39 and rK28 in detecting active VL cases and our findings indicate that rK28 polyprotein has great potential as a serodiagnostic tool. A new rK28-based RDT will prove to be a valuable asset in simplifying VL disease confirmation at the point-of-care.

## Author Summary

Visceral Leishmaniasis caused by *Leishmania donovani* is endemic in several parts of South Asia, East Africa, South and Central America. It is a vector-borne disease transmitted by bites of infected sand flies and often fatal in the absence of chemotherapy. Timely diagnosis is an essential first step in providing proper patient care and in controlling transmission. VL diagnosis in East Africa and Latin America are currently based on microscopic confirmation of parasites in tissue aspirates. The Kalazar Detect rapid test is widely used as a confirmatory test in India with very high accuracy, but sensitivity issues have severely limited its usefulness in the African sub-continent. Direct Agglutination Test is another confirmatory test used widely in East Africa and offers high sensitivity but is not field-friendly. We report on the design of a novel synthetic fusion protein capable of sequestering antibodies against three different *Leishmania donovani* antigens and the development of point-of-care tests for improving VL diagnosis. We believe the ease of use of these rapid tests and their high accuracy in detecting VL cases could make them useful as a first-line test, thereby eliminating the need for painful biopsies and ensuring better patient care.

## Introduction

*Leishmania* parasites are transmitted to mammals by the bite of female phlebotomine sand flies and occasionally by the sharing of needles, by blood transfusion, or by congenital transmission. The life-cycle of *Leishmania* has two distinct forms: the flagellated promastigotes found in the gut of the arthropod vector and non motile amastigotes, which develop intracellularly in the mammalian host. Promastigotes injected into the skin during sand fly bite are internalized by dendritic cells and macrophages in the dermis where they lose their flagella as they transform into amastigotes. They multiply and survive within the phagolysosomes through a complex host-parasite interaction [1]. The prepatent period can vary from weeks to months and during this period disease symptoms may gradually appear and worsen with disease manifestations ranging from self-healing skin lesions, to diffuse cutaneous and mucosal manifestations and, in some cases, to severe visceral involvement of the spleen, liver and lymph nodes depending on the species of *Leishmania*.

Visceral leishmaniasis, known as kala-azar (Hindi for Black fever) in the Indian subcontinent and Africa, is the most severe form of the disease affecting approximately 500,000 adults and children worldwide. Following infection, the parasites disseminate through the lymphatic and vascular systems and infect other monocytes and macrophages in the reticulo-endothelial system, resulting in infiltration of the bone marrow, hepato-splenomegaly and sometimes enlarged lymph nodes (lymphadenopathy). Mortality of VL is high in the absence of treatment, which is generally lengthy, expensive and toxic. Clinical diagnosis relies on non-characteristic symptoms (long standing fever, cachexia, anemia and hepato-splenomegaly) which are only reliable in advanced cases and in epidemic situations. Parasitological diagnosis remains the reference standard in VL diagnosis, typically undertaken by microscopic examination of Giemsa-stained bone marrow, spleen or lymph node aspirates to detect amastigotes. This is not only invasive, but also suffers from low sensitivity, requiring both highly trained laboratory personnel to biopsy patients and high-powered microscopes that are typically available only in regional clinics. Thus, tissue aspirations for routine VL screenings are not feasible as a large-scale approach in remote field areas lacking electricity and even the most basic laboratory set-up.

The development of two serological tests for diagnosis of VL, the direct agglutination test (DAT) and the rK39 strip tests, have started to circumvent the need for tissue aspirates in Sudan and the Indian subcontinent, respectively. DAT is the first-line VL serodiagnostic test employed in Sudan [2] and, despite being highly sensitive and specific [3], [4], it is not optimum as a point-of-care test as it requires a laboratory capable of holding controlled temperature with overnight incubation. The rK39 strip test became possible after the identification of the k39 kinesin gene by an immunoscreen of a *L. infantum* expression library with sera obtained from visceral leishmaniasis patients [5]. VL patients mount a strong antibody response to the 39-amino acid, tandem repeat units in the gene, and the recombinant form of this gene, rK39, has been successfully used to develop an enzyme-linked immunosorbent assay (ELISA) [6], [7] as well as a point-of-care RDT [8], [9]. The rK39 RDT is a field-friendly, easy to use format that has been extensively tested in many countries. In a WHO supported multicenter trial, the FDA-approved rK39 RDT (Kalazar Detect- Inbios, Seattle) demonstrated excellent sensitivity (>95%) and specificity (>90%) in the Indian subcontinent (India and Nepal), but only moderate sensitivity (75 to 85%) and specificity (70–92%) in East Africa (Sudan, Kenya and Ethiopia) [10]. Reasons for the suboptimal performance of the rK39 RDT in Africa are not entirely clear and have been attributed to lower antibody levels to rK39 in infected individuals.

We previously identified k9 and k26, two *Leishmania* genes coding for hydrophilic proteins, and demonstrated that VL patients mount strong and specific antibody responses against K26, which can complement rK39 in a more accurate diagnosis of human VL [11]. Specific and independent antibody reactivity to each of the three antigens rK9, rK26 and rK39 have been studied and utilized in serodiagnosis of canine VL [12]. A multi-epitope, recombinant chimeric protein for serodiagnosis of canine and human VL was evaluated by fusing *L. infantum* k9 gene with single repeat units of k39 and k26 genes. ELISA with this fusion protein provided 96% sensitivity for canine VL compared to only 82% for human VL, with 99% specificity for both human and canine control groups [13].

We sought to improve serodiagnosis of VL by developing enhanced RDT prototypes that can detect >90% of the African VL cases, thus increasing diagnostic accuracy and overcoming limitations of the current rK39 RDT. The development of an affordable, simple, sensitive and specific point-of-care diagnostic test would clearly have a major impact on detection, control and treatment of visceral leishmaniasis patients. A synthetic gene, k28, was generated by fusing multiple tandem repeat sequences of the *L. donovani* haspb1 and k39 kinesin genes to the complete open reading frame of haspb2, thereby increasing antigen epitope density while providing complementing epitopes in the resulting recombinant protein. The recombinant fusion protein rK28 was evaluated on ELISA with a panel of active human Sudanese VL cases and also used to develop two prototype point-of-care tests, the results of which are presented. To our knowledge, this is the first study describing the development of a second generation rapid test for the serodiagnosis of human VL. Our results confirm that rK28 is an excellent serodiagnostic tool that has the potential to replace rK39 protein pending larger field trials.

## Materials and Methods

### K28 gene construct

The K28 gene was synthesized at Blue Heron Biotechnology, Bothell, WA using the Genemaker technology with a six-histidine tag downstream of the N-terminal methionine. The synthetic gene includes nucleotides 142–267 encompassing three 14-amino acid (aa) repeats of (*L. donovani* haspb1 gene, GenBank accession# AJ011810.1), nucleotides 2110–2343 encompassing two 39-aa repeats of (*L. donovani* k39 kinesin protein gene, GenBank accession# DQ831678.1) and nucleotides 1–400 (the complete ORF) of *L. donovani* haspb2 gene, GenBank accession# AJ011809.1). The 795 base pair (bp) product was subcloned directionally in the Nde I/Xho I sites of pET29 (Novagen, USA), and the transformants selected in XL-10 Gold cells (Stratagene, Santa Clara, CA.). Following sequence verification of the insert, the recombinant plasmid was subsequently transformed into *E. coli* HMS-174(DE3) for expression of recombinant protein. The k28 gene sequence has been submitted to GenBank under accession number HM594686.

### Protein expression and purification

Recombinant K28 protein (rK28) was produced by growing the transformed host cell HMS-174(DE3) in 2XYS with kanamycin using a fed-batch fermentation system. The production media was inoculated with a 10% inoculum culture in a log-growth phase. The culture was grown in a 10 L bioreactor (New Brunswick Scientific, Edison, NJ) to an optical density of 8–10 (A_600_), induced with IPTG (1mM final concentration) for 2 hours, and the cells harvested by centrifugation. Cell pellets were lysed in 50 mM Tris, pH 8.0 using an 110S microfluidizer (Microfluidics, Newton, MA) and cellular debris removed by centrifugation. The supernatant containing the expressed protein was combined with Ni-NTA agarose (QIAGEN, Valencia, CA) in a batch-bind mode and incubated overnight at 4°C. The resin was then packed in a column and washed with 10 column volumes of 20 mM Tris-Cl, pH 8.0, 250 mM NaCl, 0.5% CHAPS. Bound protein was eluted with 20 mM Tris-Cl pH 8.0 containing 400 mM imidazole. The eluted fractions were diluted 1∶3 with 20 mM Tris-Cl pH 8.0 and loaded onto a Q sepharose fast flow column (GE Healthcare Biosciences, Piscataway, NJ). The peaks eluted at 200 to 300 mM NaCl were combined; ammonium sulfate was added to make the final concentration 2 M, following which hydrophobic interaction chromatography was performed using Octyl Sepharose 4 Fast Flow (GE Healthcare Biosciences). Peaks eluted at low salt concentrations were combined, dialyzed into 20 mM Tris-Cl, pH 8.0, and sterile filtered through 0.22 µm filter. The final protein concentration was determined using BCA assay (Pierce Chemical, Rockford, IL). The lipopolysaccharide content of each protein preparation was measured by a *Limulus* amoebocyte lysate test (BioWhittaker, Walkersville, MD) and shown to be below 10 endotoxin units (EU)/mg of protein.

### Patient sera for ELISA evaluation

Disease-positive sera from 62 VL patients were obtained from the Gedaref state of eastern Sudan. The inclusion criteria for VL sera samples used in this study were, (i) all patients were parasitology positive, confirmed by microscopy of lymph node or bone marrow aspirates, (ii) all patients had clinical symptoms and were diagnosed as active VL cases. Microscopic confirmation of parasites was performed by trained technologists. The median age of the patients was 10 years. 32% of the recruited patients were females and 68% were males. Panels of negative sera were from 25 healthy endemic controls (EC) from the VL endemic region of Gedaref and 20 healthy non-endemic controls (NEC) from Khartoum, Sudan. Sera from patients with other infections included malaria- (n = 10), tuberculosis- (n = 10) and Salmonella- confirmed patients (n = 10) from Khartoum, Sudan (courtesy of Dr. Sayda El-Safi, Faculty of Medical Laboratory Sciences, Khartoum University, Sudan). Sera samples used in this study were collected as a part of routine diagnosis and treatment of patients in Gedaref and Khartoum, Sudan. Patients received standard treatment for the different disease indications as outlined by the Federal Ministry of Health-Sudan. All samples were subject to appropriate ethical clearance from the Faculty of Medicine, University of Khartoum and from the National Ethical Review Board at the Federal Ministry of Health-Sudan. The entire Sudanese negative-sera panel had no past history of visceral leishmaniasis. IRB approval was not sought for this study as banked sera from IRB approved protocols were used for both the ELISA and RDT testing. No personal identifiers were used nor any clinical investigation carried out as part of this study. Information about the patient's clinical diagnosis was available to us at the time this study was undertaken. Thirty normal human sera (NHS) from U.S residents with no history of international travel were used for testing non-specific reactivity of the recombinant proteins (Equitech-Bio, Kerrville, TX).

### Antibody ELISA

rK39, rK28, rK26, rK9 were titrated (200–25 ηg/well) with different dilutions of positive and negative sera (1∶100, 1∶200, 1∶400) using a checker board titration on flat-bottomed MediSorp™ and PolySorp™ Nunc MicroWell™ plates to determine the optimized ELISA conditions. A human immunoglobulin (IgG) standard curve was constructed using chrompure human IgG (Jackson ImmunoResearch, West Grove, PA) and used as a reference standard on every plate [14]. The first two columns on every plate were coated in duplicate with 4-fold dilutions of the standard curve (100, 25, 6.25, 1.563, 0.391, 0.098, 0.024, 0 µg/well) in 0.1 M bicarbonate buffer pH 9.6, 0.01% BSA, 0.1% sodium azide. The rest of the plate was coated with 25 ηg /well of the antigen in 0.1M bicarbonate buffer pH 9.6 at room temperature for 2 hours. The non-specific reactivity on the plate was blocked with 1% BSA in phosphate-buffered saline pH 7.2, 0.1% Tween 20 for a period of 2 hours at room temperature. The plates were washed in wash buffer (PBS, 0.1% Tween 20) four times and 100 µl of 1∶400 dilution of the sera in serum diluent (0.1% BSA in phosphate buffered saline pH 7.2, 0.1% Tween 20) added to the antigen wells and 100 µl of serum diluent added to the standard curve wells in duplicates and incubated at room temperature for an hour on a micro plate shaker at 500 rpm. The plates were washed in wash buffer and the bound antibodies were assayed using 100 µl per well of 1∶10,000 diluted Rec. Protein-G HRP (Zymed, San Francisco, CA) at room temperature for 1 hour. The enzyme reaction was developed with 100 µl per well of SureBlue TMB 1-component microwell peroxidase substrate (KPL, Gaithersburg, MD) for 5 minutes. The reaction was stopped using 50 µl/well of 1 M sulfuric acid, plates were read at 450 nm on a ThermoMax microplate reader and data analyzed using SoftMax Pro (Molecular Devices, Sunnyvale, CA). The final conditions that produced the best observed test results for the Sudanese panel of sera were 25 ηg/well of antigen, 1∶400 dilution of serum, and a 1∶10,000 dilution of recombinant protein G-HRP as the enzyme conjugate for detection on Medisorp plate surface. Extensive optimization experiments were performed with different parameters in order to determine the final conditions that could best differentiate between strong positive and borderline positive VL sera from truly negative sera. Reproducibility of the ELISA results were confirmed by having at least two independent operators (blinded to the identity of test samples) perform the same assay.

### Calculations and statistical analyses

The human IgG standard curve was used as a reference standard to control for inter-plate variation, as well as to determine that the test was run properly. The standard curve was plotted on a 4-parameter curve fit, and an r^2^ value of 0.995 was required to validate the data from the plate. A receiver-operator characteristic (ROC) curve was used to evaluate all possible combinations of sensitivity and specificity and to determine an optimal cut-off that clearly discriminates between disease-positive and -negative sera [15], [16]. The ELISA test results from 105 non-VL sera {endemic controls (EC), non-endemic controls (NEC), US normal human sera (NHS) and other infection sera} were used as the negative data set, and the results from 62 VL-confirmed sera were used as positive data set. GraphPad Prism 4.0 software (GraphPad Prism Inc., San Diego, CA) was used to perform the statistical analyses. ROC curves were plotted using the software, and a table of sensitivity and specificity with all possible cut-offs were generated with 95% confidence intervals. The sensitivity of the test was determined as the fraction of the VL confirmed sera that were test positive, and specificity was calculated as the fraction of the EC, NEC, NHS and other infection patient sera that were identified to be truly test negative. The positive and negative predictive values of the tests were calculated. Area under the curve (AUC) was used as measure of diagnostic accuracy of the test providing a means to truly discriminate between disease-positive and disease-negative sera. Correlations in antibody responses were studied between individual component antigens and rK28. A nonparametric Spearman correlation was used to calculate the correlation coefficient.

### Development of rK28 rapid test formats (K28-LF and K28-DPP)

Purified rK28 was provided to two manufacturers, EASE-Medtrend (Shanghai, China) and Chembio Diagnostic Systems (Medford, NY) to develop rapid tests. The EASE-Medtrend single lateral flow test utilizes a proprietary dynamic flow principle. The test antigen is immobilized on a nitrocellulose membrane within the test zone. The liquid conjugate is applied to the device through the reagent port, priming the device to facilitate the migration of serum applied in the sample port. The specific antibodies present in the serum are captured by the immobilized antigens and subsequently visualized in the form of a magenta-colored test line by the conjugate. In the control zone, a conjugate-binding reagent is immobilized on the membrane. A magenta line in the control zone appears in every valid test. The EASE-Medtrend rK28 based prototype will be referred to as K28-LF. A subset of Sudanese VL sera with low ELISA reactivity to rK39 were selected for studying the additive effect of antigens on the lateral flow format using the K28-LF RDT.

The Chembio immunoassay format is called the Dual Path Platform (DPP). It differs from conventional lateral-flow systems in that the test sample and the marker-detecting conjugate are delivered to the test line area independent of each other. The DPP assay has two laminated strips, connected to each other as a “T” shape inside a disposable plastic cassette. The first strip receives a sample and running buffer through the sample port. The sample migrates along the strip towards the second strip containing the test and control bands. Development of the assay is achieved by adding buffer to the development port. This step releases the conjugate (colloidal gold) and facilitates its migration to the test area. Antibodies, if present in the test sample, will bind to the capture reagent immobilized on the second strip, and the conjugate will react with this complex, making the test band detectable by visual evaluation. Irrespective of the presence of antibodies in the test sample, the control band should develop to assure correct DPP assay performance. The Chembio rK28-based prototype will be referred to as K28-DPP.

### Evaluation of rK28 RDT in Sudan and Bangladesh

Testing of the two K28 RDT prototypes with larger sera panels were carried out in parallel in Sudan and Bangladesh. The rK28-DPP RDTs were tested in Sudan using 73 parasitology (LN aspirates) confirmed VL samples, 24 healthy endemic controls, 18 tuberculosis- and 20 malaria- confirmed sera. Evaluation in Bangladesh was carried out using the rK28-LF RDT and included 53 parasitologically (spleen aspirates) confirmed VL samples, 20 healthy endemic controls and 20 healthy non endemic controls. The rK39-based Kalazar Detect (InBios International Inc. Seattle, WA) RDT was used as a comparator in all studies.

Both rapid test formats use recombinant protein A-colloidal gold conjugate for detection and were performed according to manufacturer's specifications. The test results were determined in 10 minutes for the Inbios and Ease Medtrend strips and in 15–20 minutes for the Chembio DPP tests. Every sample was tested twice and the results were scored by the operator as well as independently by an individual blinded to the identity of serum samples. While microscopic confirmation of parasites is performed by well trained technologists, minimal training was required for individuals scoring the RDTs.

### Ethics statement

Sera samples used in this study were collected as a part of routine diagnosis and treatment of VL patients in Gedaref Hospital, Sudan and at the Rajshahi Medical College Hospital, Bangladesh. Study protocols for the collection were approved by the Institutional Review Boards of Khartoum University and Rajshahi Medical College. In Bangladesh and Sudan, written consent was obtained from all adult patients and parents or guardians of children. For samples from illiterate adult participants and children, verbal consent was read to and discussed with adults/guardians in presence of a literate relative, the consent form was signed by the literate relative and a fingerprint was obtained from the parent/guardian. IRB approval was not sought for this study as banked sera from IRB approved protocols were used for both the ELISA and RDT testing (retrospective study). No personal identifiers were used nor any clinical investigation carried out as part of this study. Information about the patient's clinical diagnosis was available to us at the time this study was undertaken.

## Results

### Design of the k28 synthetic gene and expression of recombinant K28 protein

**Figure 1:**
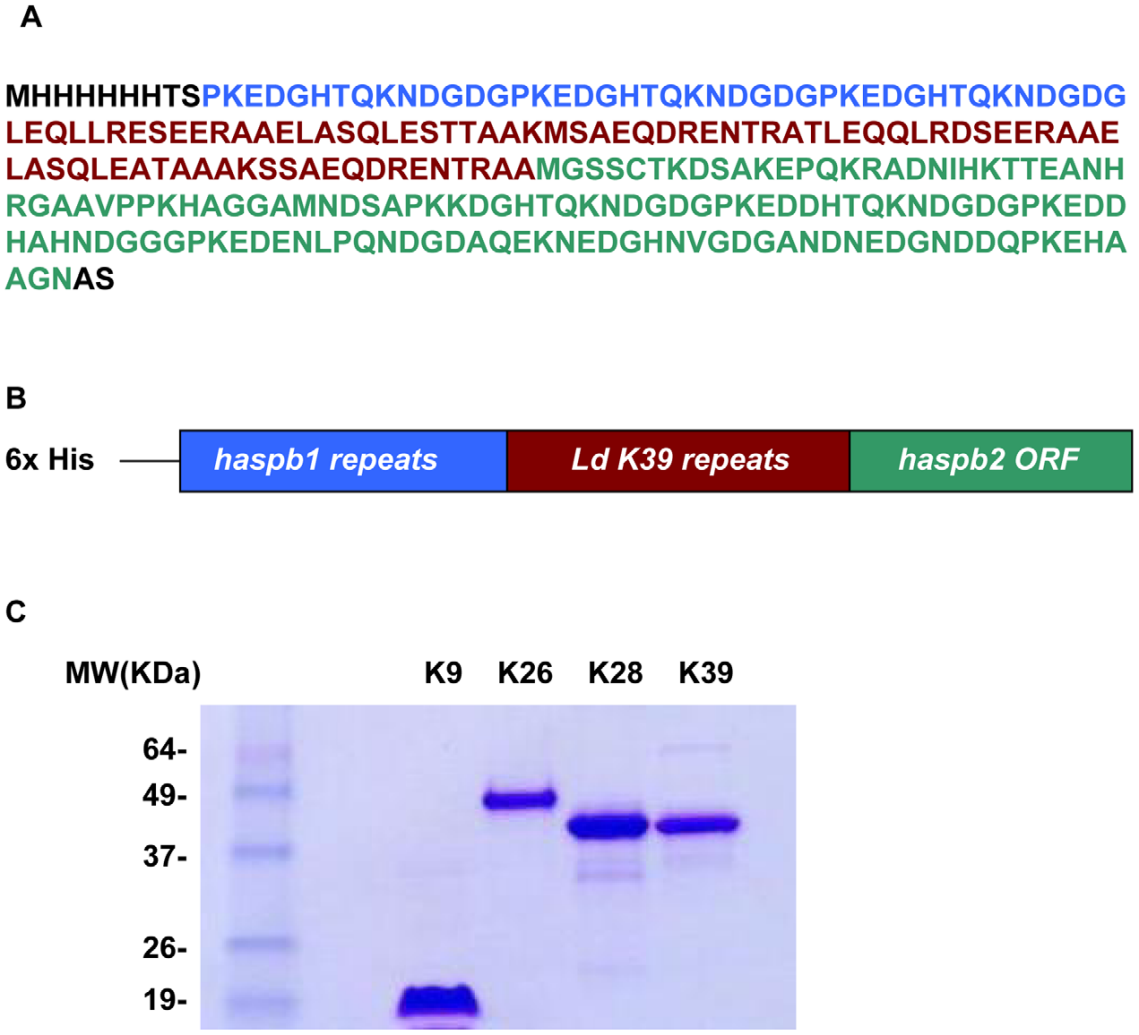
Design of the k28 gene. A: The complete amino acid sequence of the k28 gene contains an amino terminal 6 histidine tag followed by three 14 amino acid tandem repeat regions of the haspb1 gene (shown in blue), two 39 amino acid tandem repeat regions of the *L. donovani* k39 kinesin gene (shown in red) and 133 amino acid full length open reading frame of the haspb2 gene (shown in green). B: Schematic showing the rK28 fusion protein. C: 2.5 µg of purified rK9, rK26, rK28, rK39 run on a 4–22% polyacrylamide gradient gel under reducing conditions and stained with simplyblue safestain.

The synthetic gene k28 was designed by fusing nucleotide sequences for three 14-aa tandem repeats of the *L. donovani* haspb1 gene [17], two 39-aa tandem repeats of the *L. donovani* kinesin gene [18] and the entire 133 aa of the *L. donovani* haspb2 gene (Figure 1A) [17]. The 795 bp nucleotide sequence cloned in pET-29a was used to express an N-terminal 6XHis-tagged recombinant protein in *E. coli*. The fusion protein was purified by affinity chromatography over a Ni-NTA agarose matrix. The 264-aa sequence (Figure 1B) encoded an acidic protein (pI 4.73) with a predicted molecular weight of 28.33 kDa. The protein migrated aberrantly around 40 kDa on SDS-PAGE (Figure 1C). Hypothetically, the mobility of rK39 and rK28 should be slower than the mobility of rK26. The predicted molecular masses of both rK39 (35.3 kDa) and rK28 (28.33 kDa) are indeed higher than that of rK26 (26 kDa) and yet they seem to migrate faster compared to rK26 (26 kDa). The slower mobility of rK26 is due to its higher proline content compared to either rK28 or rK39.This characteristic has been observed for other proteins with high acidity and high proline/lysine content including HASPB1, K26 and K9 [11], [17], [19].

### ELISA test results with Sudanese sera on rK28, rK39, rK26, and rK9

**Figure 2:**
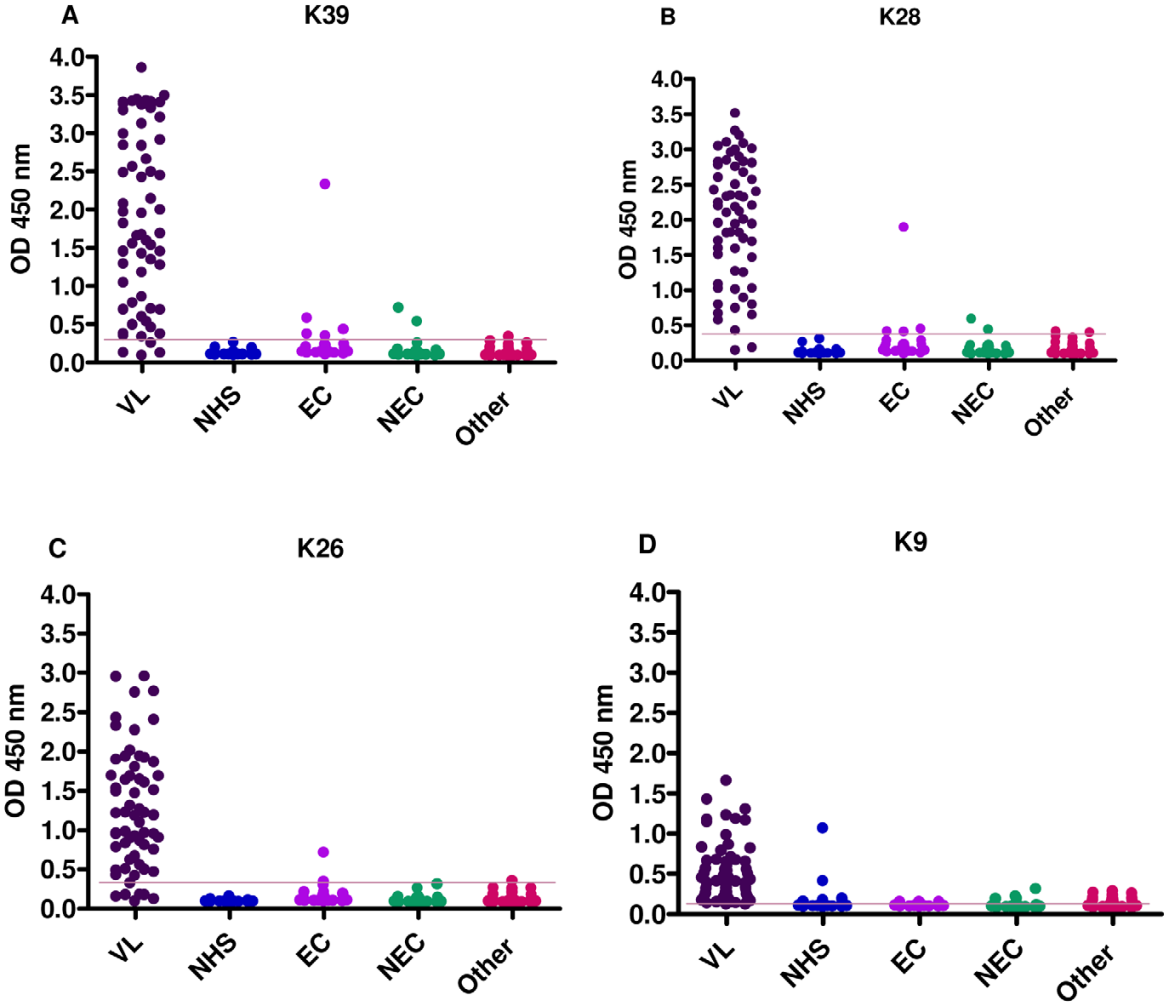
Comparison of ELISA reactivity of Sudanese sera against rK39, rK28, rK26 and rK9 proteins. Antigen specific antibody titers of individual Sudanese human sera grouped based on their clinical status. Results are expressed as the optical densities at 450 nm (A_450_). The antigens were coated at 0.25 µg/ml. A: rK39, B: rK28, C: rK26, D: rK9. The cut off values chosen from the ROC curves are shown as lines and are 0.304, 0.415, 0.315, and 0.159 for rK39, rK28, rK26 and rK9 ELISA respectively.

In order to evaluate the antigen-specific antibody responses against rK28, rK39, rK26, and rK9, antibody ELISA's were optimized to obtain the best signal-to-noise ratio and develop a reproducible and robust assay that was capable of capturing antibodies over a biologically relevant assay range. Sudanese parasitology confirmed VL-positive and -negative sera (NHS, EC, NEC and other infection sera) were tested by ELISA on rK28, rK39, rK26, and rK9 to evaluate immunoreactivities (expressed as A_450nm_ in Fig. 2) against individual proteins. The overall OD responses of individual VL-confirmed sera were quite similar for rK39 and rK28 antigens (Fig. 2A and B, respectively), and both were much higher than the responses to rK26 and rK9. The ELISA cut-off values of the 4 recombinant proteins rK28, rK39, rK26, and rK9 were 0.4151, 0.3043, 0.3149, and 0.1589, respectively, and were determined by ROC (Receiver-Operator Curve) analyses of the absorbance values at 450 nm (Table 1).

**Table 1:** ELISA (A_450_) results and diagnostic accuracy of testing Sudanese human sera on recombinant proteins.

Antigen	Cutoff (A_450)_	Sensitivity*	95% Confidence Interval	Specificity*	95% Confidence Interval	Area Under the Curve
rK39	0.3043	0.9355	84.30–98.21%	0.9238	85.54–96.65%	0.9565
rK28	0.4151	0.9677	88.83–99.61%	0.9619	90.53–98.95%	0.9839
rK26	0.3149	0.9032	80.12– 96.37%	0.9714	91.88–99.41%	0.9708
rK9	0.1589	0.9032	80.12–96.37%	0.8286	74.27–89.51%	0.9424

*Sensitivity measures the fraction of actual disease positives that are correctly identified by the test. Specificity measures the fraction of disease negatives that are correctly identified by the test.

### Diagnostic accuracy of rK28

**Figure 3:**
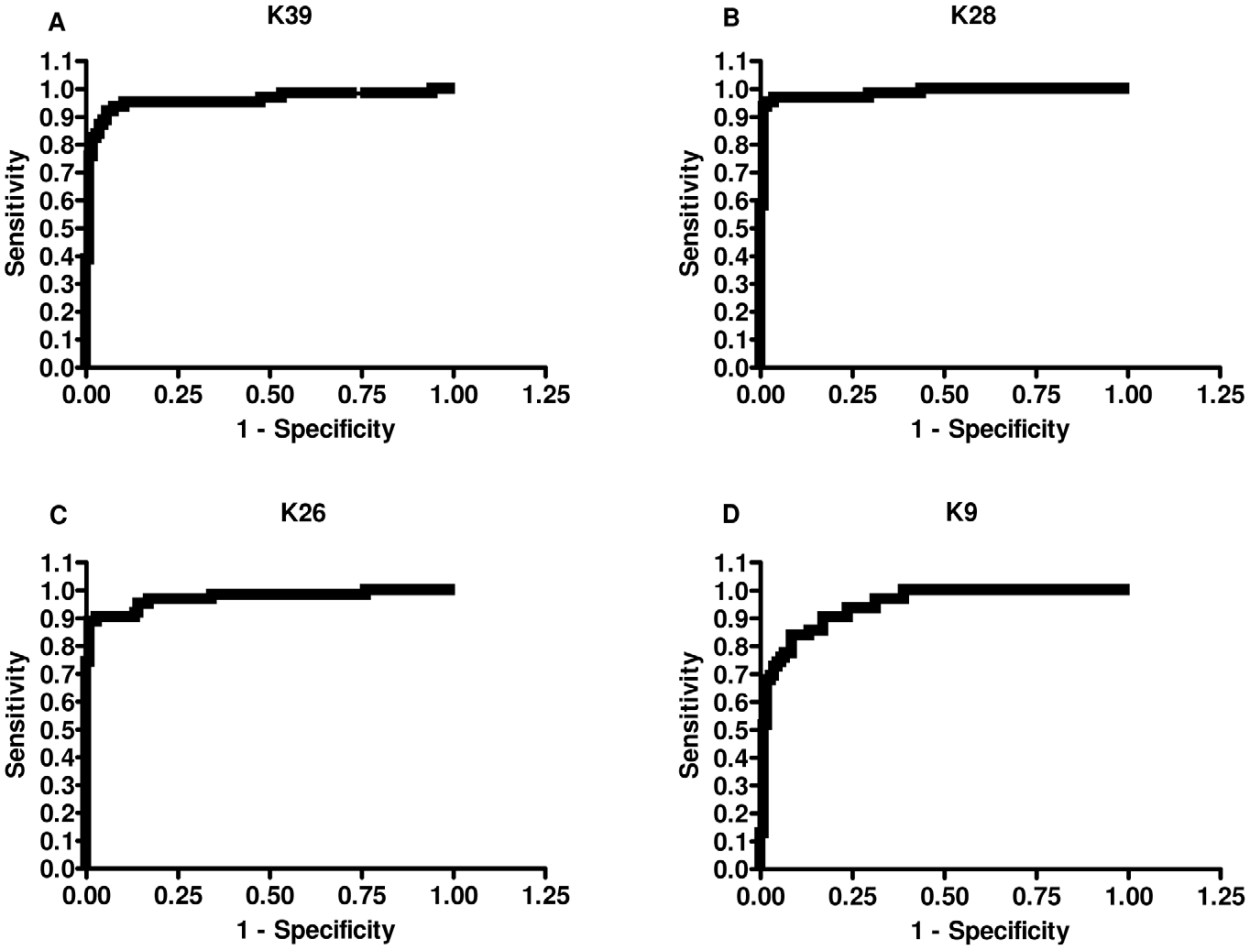
ROC curves generated from the ELISA values of Sudanese sera against rK39, rK28, rK26, and rK9. The curves were used to determine ELISA cutoff, sensitivity and specificity. A: rK39, B: rK28, C: rK26, D: rK9.

The sensitivity, specificity and area under the curve (AUC) were calculated for all 4 recombinant proteins (Table 1). rK28 was test-positive on 60 of the 62 VL-positive serum samples, yielding a sensitivity of 96.8%, while rK39 had a sensitivity of 93.5% (58/62). rK26 and rK9 both missed 6 out of 62 VL positive sera and had sensitivities of 90.3%. Specificity was calculated using a panel of 105 sera that included healthy endemic, healthy non-endemic, and other confirmed infectious disease sera, together with sera from healthy non-travelers from the United States. Based on the ELISA cut-off (Table 1), rK26 had the highest specificity of 97.1%, closely followed by rK28 with a specificity of 96.2%. rK39 had a specificity of 92.4% while the least specific was rK9 with a specificity of 82.9%. All healthy U.S donors (30 samples) were test-negative on rK39, rK28 and rK26 ELISA. rK9 was least specific, as 4/30 U.S donors reacted non-specifically on the ELISA (data not shown). The area under the curve (AUC) is a widely accepted metric for evaluating diagnostic accuracy [20]. The greater the AUC, the better the accuracy of the diagnostic test, and an AUC of 1 represents perfect accuracy [21]. The ROC curves obtained for the ELISA using absorbance values for rK28, rK39, rK26 and rK9 are shown in Figure 3. rK28 had the highest AUC (AUC_rK28_) with a value of 0.98. This was followed in order of accuracy by AUC_rK26_ = 0.97, AUC_rK39_ = 0.96, and finally AUC_rK9_ = 0.94.

### Benefits of the cumulative effect of rK28 in low reactive K39 VL serum

**Figure 4:**
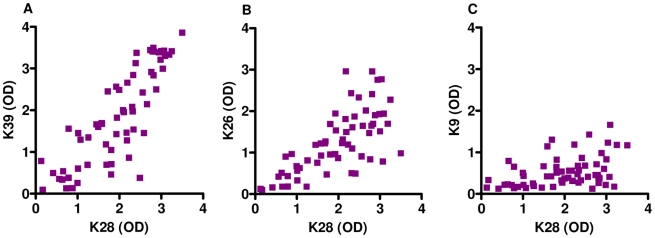
Correlations in serum responses between rK28 and the individual component proteins rK39, rK26 and rK9 on testing with Sudanese VL patient sera. Correlation coefficients were calculated using ELISA results of sera from 62 individuals with confirmed VL. A: Comparison of antibody levels (OD) against rK28 and rK39, B: Comparison of antibody levels (OD) against rK28 and rK26, C: Comparison of antibody levels (OD) against rK28 and rK9. Sudanese VL patients with extremely low rK39 antibody titers demonstrate much higher titers against rK28 as demonstrated by a strong and statistically significant positive correlation on the Spearman test (r = 0.8383, p<.0001).

**Figure 5:**
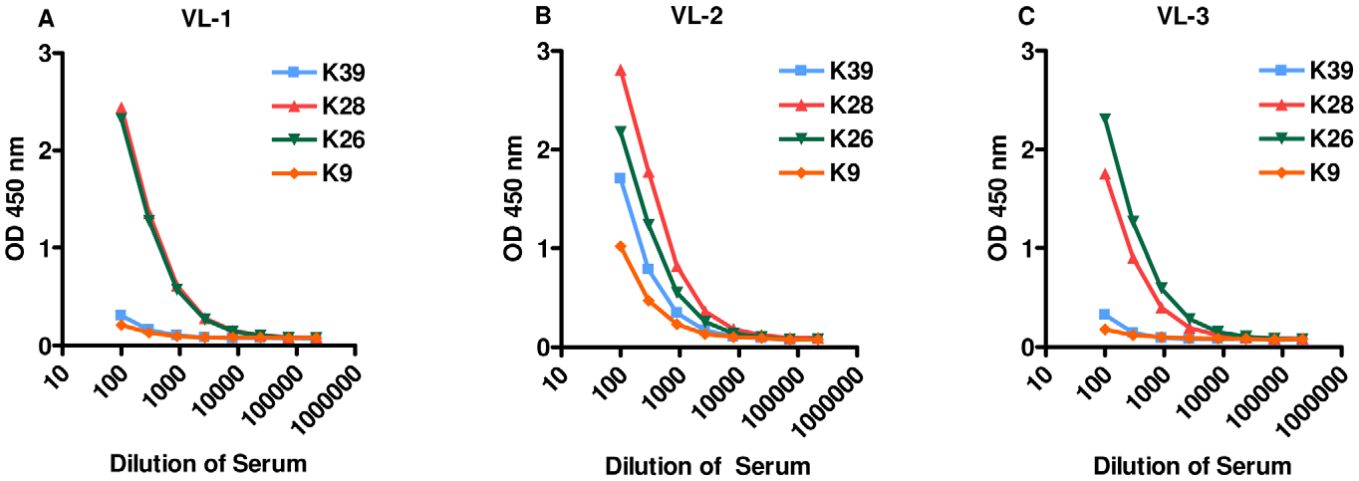
Serum titrations of low rK39 reactive VL-confirmed patient sera evaluated on ELISA. A_450_ values show a cumulative increase in serum responses to rK28 polyprotein compared to proteins rK39, rK26 and rK9. A: VL-confirmed sera from patient 1; B: VL-confirmed sera from patient 2; C: VL-confirmed sera from patient 3.

rK28 is a fusion polyprotein comprising regions of *L. donovani* haspb1 (*L. infantum* k26 homologue), *L. donovani* kinesin (*L. infantum* k39 homologue) and *L. donovani* haspb2 (*L. infantum* k9 homologue). For many of the Sudanese VL sera tested, the relative absorbance observed were higher on the rK28 ELISA compared to rK39, rK26 or rK9. We also observed a subset of individual sera with very low reactivity to rK39, but much higher reactivity to rK28. The absorbance values (A_450_) of individual sera to rK28 were plotted against the absorbance values of the three individual proteins rK39, rK26 and rK9 and are shown as scatter plots (Figure 4). A nonparametric spearman correlation analysis was done to calculate the correlation coefficient r and two-tailed P values. The antibody levels measured by rK39 and rK28 (Spearman r = 0.8383, P<0.0001); rK26 and rK28 (Spearman r = 0.7141, P<0.0001); rK9 and rK28 (Spearman r = 0.4112, p = 0.0009) displayed a positive and significant correlation. Overall, there seemed to be a cumulative increase in the absolute magnitude of the antibody responses when using rK28 protein to capture serum antibodies. In order to investigate the cumulative effects of rK28, VL sera with low K39 reactivity were selected, and the responses against individual proteins titrated against 10-fold serial dilutions of the sera (1∶100–1∶1000000) in an ELISA (Figure 5). We observed that a majority of the VL sera tested in this manner had a much higher response to rK28 and in some cases a stronger response to rK26 (Figure 5C).This may be due to the fact that rK28 can capture circulating antibodies against all three component proteins (Haspb1, LdK39, and Haspb2) leading to a more robust signal. Therefore, it is likely that tests using rK28 protein could potentially diagnose individuals that are missed by rK39.

### Development of K28-LF RDT and prototype testing in Bangladesh

In order to evaluate rK28 on a point-of-care test, single lateral flow RDT prototypes (K28-LF) were developed by EASE MedTrend (Shanghai, PRC) based on their proprietary dynamic flow principle. Preliminary screening of 13 VL Sudanese sera (selected on the basis of low rK39 ELISA reactivity) demonstrated a much higher sensitivity for the K28-LF (92.3%) compared to a much lower sensitivity with Kalazar Detect (69.2%) (Table 2). Neither RDTs gave any false positive results with sera from U.S healthy subjects. To obtain a more realistic performance of the RDTs in a VL endemic region, we next evaluated both the rK28-LF and Kalazar Detect on a larger panel of VL confirmed sera in Bangladesh. 53 parasitology confirmed VL sera and 40 healthy endemic control sera with no history of VL were evaluated. Once again the K28-LF RDT provided more favorable results (98.1% sensitivity, 92.5% specificity) compared to Kalazar Detect (88.7% sensitivity, 100% specificity) (Table 3).

**Table 2:** Results of weak rK39-reactive Sudanese VL sera on K28-LF and Kalazar Detect RDTs.

Sudan Sera	% Sensitivity	95% Confidence Interval	% Specificity	95% Confidence Interval
K28-LF*	92.3	63.97–99.81%	100	88.43–100.0%
Kalazar Detect*	69.2	38.57–90.91%	100	88.43–100.0%
No. of Sera Tested	13		30	

*K28-LF is manufactured by Ease-Medtrend, and the Kalazar Detect is manufactured by Inbios International, Inc. A panel of US healthy sera was used for specificity testing.

**Table 3:** Results of testing human VL sera from Bangladesh on K28-LF and Kalazar Detect RDTs.

Bangladesh Sera	% Sensitivity	95% Confidence Interval	% Specificity	95% Confidence Interval
K28-LF*	98.1	89.93–99.95%	92.5	79.61–98.43%
Kalazar Detect*	88.7	76.97–95.73%	100	91.19–100.0%
No. of Sera Tested	53		40	

*K28-LF is manufactured by Ease-Medtrend and the Kalazar Detect test is manufactured by Inbios International, Inc. All VL patient sera were confirmed by splenic aspirates. Sera from healthy endemic individuals were used as controls for specificity testing.

### Development of rK28-DPP RDT and prototype testing in Sudan

A second prototype test of rK28 (K28-DPP) using a distinct technology was developed by Chembio Diagnostic systems. Sera from 73 parasitology confirmed VL patients who were DAT or smear-positive and 62 negative sera (24 endemic controls, 20 malaria- and 18 tuberculosis-confirmed patients) with no history of VL were evaluated in Sudan. All sera were also tested with Kalazar Detect as a comparator (Table 4). The K28-DPP RDT proved to be superior and provided a sensitivity of 95.9% and specificity of 100% while the Kalazar Detect yielded a sensitivity of 86.3% and specificity of 96.4%. The DAT, which was performed on every serum sample used in this study, provided a sensitivity of 94.5% and specificity of 100%.

**Table 4:** Results of testing human VL sera from Sudan on K28-DPP and Kalazar Detect RDTs.

Sudan Sera	% Sensitivity	95% Confidence Interval	% Specificity	95% Confidence Interval
K28 DPP*	95.9	88.46–99.14%	100	94.22–100.0%
Kalazar Detect*	86.3	76.25–93.23%	98.4	91.34–99.96%
DAT*	94.5	86.56–98.49%	100	94.31–100.0%
No. of Sera Tested	73		62	

*K28 DPP is manufactured by Chembio Diagnostics Systems Inc., the Kalazar Detect test is manufactured by Inbios International Inc, and DAT is the Direct Agglutination Test. Sera from healthy endemic individuals were used as controls for specificity testing.

## Discussion

Aspirates from bone marrow, lymph nodes or the spleen are typically done to confirm the diagnosis of visceral leishmaniasis. Although the specificity is high, the sensitivity of microscopy varies and is greatly influenced by the experience of the individual making the smear, the quality of the smear, and the reagents used. Microscopy is available only in tertiary care or referral centers/hospitals in endemic countries and is a time-consuming procedure. These factors make it difficult to accurately diagnose VL patients in primary care settings. The identification of rK39 as a marker of active VL disease [5] followed by its use in a rapid test format [9] has revolutionized VL diagnosis in the Indian subcontinent. While the rK39-based rapid test has greater than 95–98% sensitivity in the Indian subcontinent and is now widely used as a means of confirming diagnosis of VL patients, its sensitivity is lower in the VL endemic regions of Africa, limiting its usefulness as a widely used point-of-care serodiagnostic test. Detailed studies of human leukocyte antigen (HLA) polymorphisms between VL subjects from the Indian and African subcontinents would be valuable to explain differences in epitope specificities between these populations.

This study was initiated with the goal of developing a highly sensitive, specific, cost-effective, and rapid point-of-care serodiagnostic test for VL diagnosis that would improve upon the rK39 RDT. Our strategy included design of a synthetic gene, k28, harboring sequences fused from three *L. donovani* tandem repeat containing genes (*haspb1*, *LdK39 and haspb2*). Previous work done by our group revealed that increasing the number of tandem repeat units exponentially increases the ability to capture antibodies in the serum [22]. We utilized this information and incorporated multiple tandem repeat regions of *haspb1* and *LdK39* in order to increase the antigen epitope density within the resulting fusion protein. The *L. infantum* homologues of these genes have previously been shown to have good serodiagnostic value for both human and canine VL [11], [12], [13]. The tandem repeat regions found in many protozoan proteins usually contain immunodominant B-cell epitopes capable of generating high levels of antibody response in infected individuals [23], [24], [25], [26].

Serological responses of Sudanese VL patients were tested by ELISA against rK28 and compared with individual *L. infantum* homologue proteins rK39, rK26, and rK9. In order to model a realistic scenario in a VL endemic country, our specificity data included healthy endemic and non-endemic control sera as well as sera from individuals with other diseases. Healthy human sera from the USA were also included as part of the specificity panel to evaluate if the recombinant proteins had any false positive reactivities. From the testing done on ELISA, rK28 was more sensitive and specific than rK39. The ROC curves also predicted a higher diagnostic accuracy for rK28 compared to rK39.

We next sought to determine whether VL sera with low/borderline ELISA reactivity against rK39 could be detected with greater accuracy using rK28. Our results showed that many of the rK39 low-reactive sera had higher reactivity to rK28 and in some instances to rK26. The benefit of using rK28 for VL diagnosis arises from its ability to capture circulating antibodies to 3 *Leishmania* antigens compared to rK39, which binds antibodies specific to a single antigen. The cumulative antibody binding observed with rK28 raises the intensity of the signal and makes the border-line positive low rK39 sera distinctly positive.

The higher sensitivity of rK28 in effectively identifying low rK39 sera prompted us to exploit this characteristic for developing rK28-based point-of-care rapid tests. rK28 was provided to two independent manufacturers for prototype RDT development to ensure that test format-specific constraints would not limit product development. Also, having multiple manufacturers creates a healthy competition promoting lower costs and better quality tests for clinicians.

Testing of 13 low rK39 reactive Sudanese VL sera with the rK28-LF prototype confirmed significantly higher sensitivity (92%) afforded by a rK28-based test in comparison to the Kalazar Detect test (69%). To evaluate accuracy of the rK28 RDT prototypes in detecting VL patients, independent studies were conducted with larger sera samples in Sudan and Bangladesh, two countries where VL is endemic. As the prototype tests were manufactured on a small scale for conducting pilot studies, the two RDT prototypes could not be tested in both countries. The rK28-DPP afforded 96% sensitivity in detecting DAT or smear-positive active VL patients in Sudan, while the rK28-LF RDT provided 98% sensitivity in detecting microscopy-confirmed active VL cases in Bangladesh. Overall, both rK28-based prototype tests proved more sensitive in detecting VL cases compared to the rK39-based tests. rK28-DPP tests also proved to be highly specific (100%) in Sudan while the rK28-LF was somewhat less specific (92.5%) in Bangladesh. Large-scale field studies in both countries for selection of the final test format are planned. Further testing in the field and close follow-up of healthy individuals in VL endemic areas who have tested positive on the rK28 tests, but lack clinical symptoms, will further illustrate characteristics of the fusion protein and help us determine whether rK28 is capable of acting as an early marker of infection. The samples used as a part of this study were VL patients confirmed by parasitology, therefore, the role of K28 rapid test in detecting parasitology negative and DAT negative VL patients is yet to be studied. This will be crucial in determining the true accuracy of the rapid tests.

Due to the lack of accurate and non-invasive field-applicable tests, VL patients (a majority of whom are children) undergo extreme pain and discomfort as a result of diagnosis by tissue biopsy. The K28 RDT's could become a crucial tool for VL diagnosis, providing an easy alternative to biopsies. Early diagnosis and treatment of VL are crucial for both the affected individual and for the community. Untreated VL patients act as a reservoir of disease, especially in Africa and the Indian subcontinent where the disease is anthroponotic. Early and accurate case detection and treatment are essential components in VL control and elimination programs. Identification of affected individuals using an affordable serodiagnostic test prior to using expensive confirmatory tests for parasite detection and subsequent initiation of treatment would greatly impact timely case management and disease control. Use of a low-risk, field-based diagnostic test to detect active disease with greater accuracy, as well as monitor sub-clinical infection rates would significantly impact population-based control of disease and potentially reduce time to cure for individual patients. Multicenter large scale field evaluation of these prototype formats, including the rK39-based RDT as a comparator, are being planned to enable selection of a rK28-based RDT.

In conclusion, we have designed a new synthetic fusion protein for improved serodiagnosis of VL. The rK28 protein affords higher sensitivity in detecting active VL cases compared to rK39 both on ELISA and RDT format. The development of an rK28-based point-of-care test has yielded promising results and will become a valuable tool in rapid diagnosis of VL in conjunction with complementary tools such as parasite circulating antigen detection tests and nucleic acid detection tests and permit addressing the under-reporting of this neglected disease.

## Supporting Information

Checklist S1 STARD Checklist
(0.06 MB DOC)

Flowchart S1 Study Design for Diagnostic Accuracy (STARD) Flow Diagram
(0.60 MB TIF)
