# Supplementary figures and images for: Design, Development and Evaluation of rK28-Based Point-of-Care Tests for Improving Rapid Diagnosis of Visceral Leishmaniasis
Source: PLoS Negl Trop Dis. 2010 Sep 14;4(9):e822. doi: 10.1371/journal.pntd.0000822 (PMC2939046; doi:10.1371/journal.pntd.0000822)

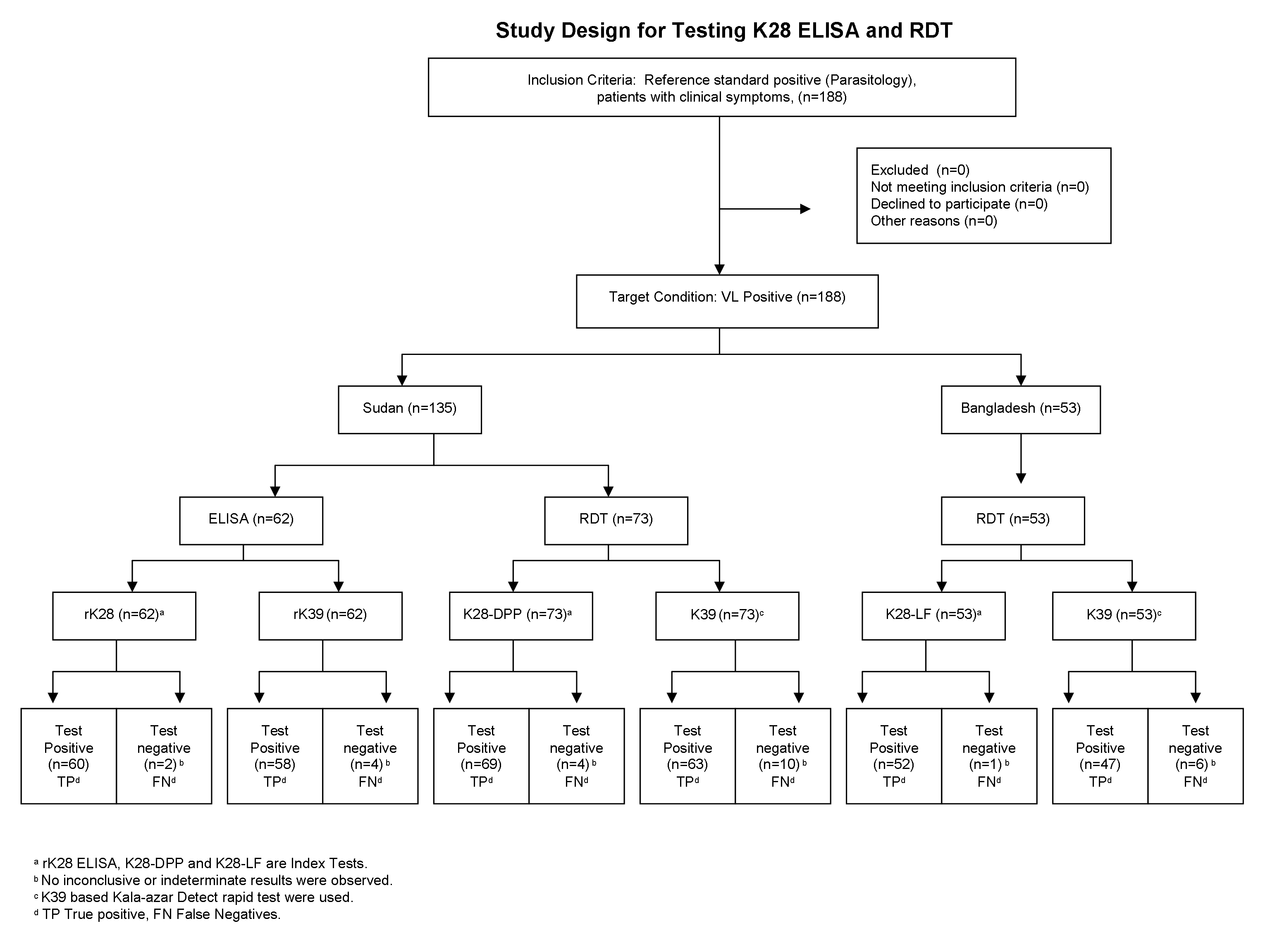

Supplement: Flowchart S1 — Study Design for Diagnostic Accuracy (STARD) Flow Diagram (0.60 MB TIF) [file pntd.0000822.s002.tif]
